# 造血干细胞移植相关血栓性微血管病的诊疗进展

**DOI:** 10.3760/cma.j.issn.0253-2727.2021.08.018

**Published:** 2021-08

**Authors:** 丽萍 杨, 晓 刘, 晓辉 张

**Affiliations:** 北京大学人民医院、北京大学血液病研究所 100044 Peking University People's Hospital, Peking University Institute of Hematology, Beijing 100044, China

造血干细胞移植相关血栓性微血管病（TA-TMA）是一种造血干细胞移植（HSCT）后的严重并发症，其发生率为0.5％～76％，死亡率为50％～90％[1]–[3]。TA-TMA是以微血管内皮细胞损伤促进微血栓形成为主要病理生理机制的多系统疾病[4]。TA-TMA与传统血栓性微血管病如非典型溶血尿毒综合征（aHUS）和血栓性血小板减少性紫癜（TTP）具有相似的病理生理特征和临床表现，但是其发病机制不同于aHUS和TTP，临床预后差异较大[3],[5]–[6]。TA-TMA诊断缺乏统一标准。目前认为，病理诊断是TA-TMA诊断的金标准。现就近年来TA-TMA相关研究综述如下，主要聚焦于TA-TMA诊断标准及治疗方案。

一、TA-TMA发病机制

TA-TMA发病机制尚不明确，目前主要认为与多种因素引起的血管内皮细胞损伤有关，如预处理方案、免疫抑制剂应用、补体系统异常、感染和移植物抗宿主病（GVHD）等[7]。炎症因子（IL-1、IL-8、TNF-α等）、血栓调节蛋白和中性粒细胞胞外诱捕网（NET）等参与TA-TMA内皮损伤的发病机制（表1）[8]。近年来，补体系统异常受到越来越多学者的关注[2],[9]–[12]。TA-TMA患者血浆中存在补体因子H（CFH）相关基因缺失和CFH抗体，表明补体旁路途径参与TA-TMA内皮损伤的发病机制[13]。补体片段C4d是补体经典途径激活的重要产物，C4d在肾脏和其他脏器沉积表明，补体经典途径与TA-TMA发病相关[14]–[15]。补体膜攻击复合物（C5b-9）升高与TA-TMA的不良预后有关，证实补体异常活化在TA-TMA的发病机制中发挥重要作用[3]。根据“二次打击学说”[8]：在预处理和移植后早期阶段，正常内皮细胞在放化疗、长期制动、无关供者、HLA不匹配等危险因素作用下，形成内皮促凝状态，为“一次打击”；在移植后造血重建阶段，促凝状态的内皮细胞在钙调磷酸酶抑制剂和雷帕霉素靶蛋白抑制剂、GVHD和感染等危险因素作用下，造成内皮细胞损伤，为“二次打击”，继而形成微血管血栓，最终导致TA-TMA发生。

**表1:** 造血干细胞移植相关TA-TMA内皮细胞标志物及补体异常相关参数[7],[10],[16]–[17]

内皮细胞标志物	补体
VEGF	C3
NO	C5b-9
PAI-1	C4d
CEC	CFH
TF	CFHR1
E-selectin	CFHR3
ICAM-1	CFHR4
PECAM-1	CFHR5
IL-1	CFI
IL-8	CFB
IFN-γ	THBD
NET	Ba
TNF-α	CD46或MCP
	CD55

注：VEGF：血管内皮生长因子；NO：氮氧化合物；PAI-1：纤溶酶原激活剂抑制物-1；CEC：循环内皮细胞；TF：组织因子；E-selectin：E-选择素；ICAM-1：细胞间黏附分子-1；PECAM-1：血小板内皮细胞黏附分子-1；NET：中性粒细胞胞外诱捕网；CFH：补体因子H；CFHR：补体因子H相关基因；CFI：补体因子I；THBD：血栓调节蛋白；MCP：补体膜辅助调节蛋白

二、TA-TMA的诊断标准

1. TA-TMA临床特征：TA-TMA是一种累及多系统的疾病，主要临床特征是微血管溶血性贫血、血小板减少、乳酸脱氢酶（LDH）升高、破碎红细胞增多、严重高血压、蛋白尿和血浆sC5b-9水平升高等[3],[6]。破碎红细胞增多提示微血管内血栓形成，导致在剪切力作用下红细胞受损，对诊断TA-TMA有重要意义。然而，破碎红细胞不仅发生于TA-TMA，还可见于DIC、溶血性贫血和TTP等，但破碎红细胞比例常低于1％。TA-TMA患者常因肾功能受损及红细胞破坏，表现为LDH升高。但LDH的特异性较差，容易受心肌、肝脏疾病及骨骼肌肉疾病等影响。红细胞破坏过多亦可引起结合珠蛋白下降，Coombs试验阴性可排除自身免疫性溶血性贫血，但二者特异性均不高。TA-TMA多累及肾脏，持续增加的蛋白尿通常提示肾脏损害。蛋白尿/肌酐比值已取代24 h蛋白尿，成为评估肾功能的重要方法[18]。

2. TA-TMA的组织学病理特征：TA-TMA微血栓发生于几乎所有脏器，其中肾脏受累最为常见，胃肠道、肺、脑及心脏等均可受累。病理诊断是TA-TMA诊断的金标准。

（1）肾脏TA-TMA：肾脏是TA-TMA最常累及的器官，临床表现为肾小球滤过率（GFR）下降、肌酐升高、高血压和蛋白尿[3],[6],[19]。移植后患者尸检或肾脏活检发现，肾脏TA-TMA发生率为20％～46％[20]–[22]。Laskin等[15]分析肾组织C4d染色的移植后患者样本（尸检或活检）发现，肾脏TA-TMA患者肾小动脉C4d沉积明显增多，肾小管周围毛细血管和管状基底膜C4d沉积较少见且仅见于TA-TMA组，而两组均存在肾小球C4d沉积，但无明显差异。因此，肾小动脉C4d沉积可作为TA-TMA的组织学特征性表现。肾脏TA-TMA组织学表现：①肾小血管血栓[15],[20],[23]–[24]；②肾小球间质溶解[15],[20],[23]–[25]；③毛细血管袢阻塞[15],[20],[23]–[25]；④系膜破碎红细胞[22]–[25]；⑤肾小球毛细血管壁增厚[22]–[25]；⑥基底膜双线征[22]–[25]；⑦小动脉透明样变性[22]–[23]；⑧特征性的肾小动脉C4d沉积[15],[26]–[27]等。

（2）肠道TA-TMA（iTMA）：胃肠道是TA-TMA常累及的脏器，其发生率仅次于肾脏[24]。iTMA临床表现为腹痛、腹泻和呕吐等，其诊断依据临床表现、实验室检查和肠组织活检等[28]。2004年，Nishida等[29]首次提出肠道是TA-TMA的靶器官，对于移植后难治性腹泻患者，临床需鉴别iTMA和肠道aGVHD。多项研究显示，并非所有组织学诊断为iTMA的患者均满足TA-TMA的临床诊断，提示肠道活检能够辅助诊断TA-TMA[28],[30]–[31]。然而，有学者认为iTMA不同于TA-TMA，是一种单独的移植后疾病[31]。但应注意，TA-TMA是一种累及多器官内皮损伤的疾病，iTMA只是TA-TMA在肠道受累的表现。肾脏TA-TMA可累及肾脏和肠道，iTMA可累及除肾脏和肠道之外的其他部位。肠道aGVHD是iTMA的危险因素[24]。Jodele等[6]提出iTMA的诊断标准：临床表现为剧烈腹痛、胃肠道出血及临床性肠梗阻；影像学检查显示肠梗阻征象及黏膜增厚；胃肠镜检查显示黏膜侵蚀及出血；组织学特征为腺体消失/黏膜梗死、黏膜出血/红细胞溢出到组织、管腔内破碎红细胞和纤维素样坏死、可有血管内血栓形成、内皮细胞肿胀和分离及黏膜完全脱落等。

TA-TMA的临床诊断标准很容易低估TA-TMA的发生率，而肠道活检能够辅助诊断TA-TMA。iTMA的组织学表现如下：①血管周围黏膜出血[28],[30]–[34]；②血管内皮细胞肿胀[24],[30],[32]–[36]；③内皮细胞剥离[24],[28],[30]–[34]；④内皮细胞凋亡[30]–[31],[34]；⑤毛细血管管腔内破碎红细胞[24],[30],[32]–[35]；⑥管腔内纤维素沉积/纤维素样坏死[24],[29],[31]–[33],[35]–[36]；⑦管腔内微血栓沉积[24],[29]–[36]；⑧黏膜完全脱落[24],[28],[31]–[33],[36]；⑨腺体消失[30]–[34]。iTMA和GVHD若同时发生，仅依据临床表现难以鉴别，病理组织学鉴别尤为重要。

（3）其他临床及组织学表现：尽管TA-TMA可直接累及中枢神经系统（CNS）血管，但临床上TA-TMA相关的CNS损伤主要表现为急性高血压引起的CNS出血，临床表现为精神错乱、头痛、幻觉、视觉障碍、癫痫等[6],[16],[37]。若不及时有效控制高血压，易进展为可逆性后部脑病综合征（PRES）。PRES是一种急性脑病综合征，临床表现为头痛、癫痫、精神错乱、视觉障碍等，影像学表现为皮层下血管源性水肿[38]。TTP多伴有神经精神症状，而TA-TMA出现神经精神症状的研究报道相对较少。TA-TMA可累及肺小动脉导致肺动脉高压，临床表现为低氧血症、气促、呼吸困难等[39]–[41]，组织学表现为内皮细胞受损、剥脱，微血栓形成，及破碎红细胞外渗至肺间质[39]。右心室高压是肺TA-TMA的危险因素，超声心动图可作为考虑肺TA-TMA的常规筛查手段[41]–[42]。广泛血管内皮细胞损伤可引发多发性浆膜炎，临床表现为心包积液、胸膜腔积液及腹水（不伴发全身水肿）等，但其发病机理尚未明确[6]。

3. 诊断标准：TA-TMA的诊断标准包括BMT-CTN[1]、IWG[43]、Cho等[44]、City of Hope[45]和Jodele等[6]提出的诊断标准（表2）。BMT-CTN标准是在George等[46]对35篇研究中28种诊断标准综述的基础上，纳入其中最常用的4种诊断特征[1]（详见表2）。IWG工作小组[43]认为，引起肾功能不全病因复杂，TA-TMA患者神经异常的发生率较TTP明显少见。因此，IWG标准并未将肾功能或神经系统异常纳入到诊断标准中。IWG标准与BMT-CTN标准的主要区别在于前者破碎红细胞超过4％，后者包含肾功能/神经损害。Cho等[44]研究发现囊括伴发的肾功能和神经系统异常可能容易漏诊疑似TA-TMA病例，因此其在BMT-CTN标准基础上，除外伴发的肾功能或神经损害该项。City of Hope标准较为少用[45],[47]，其肌酐和LDH指标均不同于之前的标准，且破碎红细胞只需要存在即可满足诊断。Jodele等[6]首次将组织学证据、高血压、蛋白尿及sC5b-9水平升高纳入TA-TMA的诊断标准，并且不要求破碎红细胞具体数目/比例，只需存在即可，此点与City of Hope标准相同。组织学活检有微血栓证据或满足表2所列实验室或临床指标中的5项，即可满足TA-TMA的诊断[6],[48]。近年来，Jodele等提出的诊断标准受到国内外学者们的广泛认可[9],[49]–[53]。

**表2:** 造血干细胞移植相关血栓性微血管病（TA-TMA）不同时期的诊断标准

诊断标准	发表年份	破碎红细胞	LDH升高	血小板减少^a^	贫血^b^	Coombs试验阴性	血清结合珠蛋白下降	无法解释的肾脏^c^或神经系统异常	凝血功能正常	sC5b-9升高
BMT-CTN[1]	2005	≥2/HPF	是	−	−	是	−	是	−	−
IWG[43]	2007	≥4％（8/HPF）	是	是	是	−	是	−	−	−
Cho等[44]	2010	≥2/HPF	是	是	是	是	是	−	是	−
CityofHope[45]	2013	存在	>2倍正常值上限	是	−	−	−	肌酐>1.5倍基线值	−	−
Jodele等[6]	2015	存在	是	是	是	−	−	蛋白尿≥300 mg/L，高血压	−	是

注：HPF：高倍镜视野；Scr：血清肌酐；^a^血小板计数低于50×10^9^/L或降幅超过50％；^b^血红蛋白下降或红细胞输注需求增加；^c^血清肌酐超过基线值的2倍或肌酐清除率低于基线值的50％

高血压、蛋白尿和LDH升高在TA-TMA诊断前即可发现，可作为早期诊断指标指导临床早期干预，以改善患者预后[3]。HGB<80 g/L、随机尿蛋白>300 mg/L、随机尿蛋白/肌酐比值>2 mg/mg和补体sC5b-9升高与TA-TMA的预后不良有关，尤其当蛋白尿和sC5b-9升高，TA-TMA预后极差（1年总生存率<20％）[3]。

高危TA-TMA（hrTA-TMA）或重度TA-TMA是在满足TA-TMA诊断标准的基础上，合并蛋白尿≥300 mg/L（或随机尿蛋白/肌酐≥2 mg/mg）和血浆sC5b-9升高（≥244 µg/L）[2],[32]–[33]。

根据TA-TMA确诊时间，TA-TMA可分为早发型（发生于移植后100 d内）和迟发型TA-TMA（发生于移植后100 d以后）[54]–[55]。早发型TA-TMA患者血浆中钙调磷酸酶抑制剂水平较高，且多伴发急性GVHD；迟发型TA-TMA患者易并发慢性GVHD[55]。

三、TA-TMA的治疗

TA-TMA的一线治疗以支持治疗为主，包括减停钙调磷酸酶抑制剂和雷帕霉素靶蛋白抑制剂、控制高血压以及治疗感染和GVHD等可能会诱发TA-TMA的合并症[6],[8]。血浆置换（TPE）、依库珠单抗（eculizumab）、利妥昔单抗（rituximab）和去纤苷（defibrotide）等有一定疗效。

1. 依库珠单抗：依库珠单抗是一种人源型抗C5单克隆抗体，临床常用于治疗aHUS和阵发性睡眠性血红蛋白尿症（PNH），可通过阻断补体膜攻击复合物C5b-9的形成阻止内皮和组织损伤，目前其治疗TA-TMA有效的依据越来越多[9],[56]–[57]。2013年，Peffault等[58]首次将依库珠单抗用于治疗一例TA-TMA患者，获得缓解。依库珠单抗治疗TA-TMA的中位缓解率为67％（50％～93％）（表3）[56],[59]–[61]。Jodele等[9]对64例hrTA-TMA患者使用依库珠单抗治疗，64％的患者获得缓解，移植后一年总生存率为66％。血清sC5b-9水平与依库珠单抗剂量和恢复时间呈正相关。补体sC5b-9≥244 µg/L的成年患者，依库珠单抗推荐起始剂量为每3天900 mg，sC5b-9恢复正常、血清总补体（CH50）<10％后改为诱导剂量，每周900 mg，维持4周。依库珠单抗每次用药前检测血清依库珠单抗浓度和CH50，每周2次检测sC5b-9浓度。若依库珠单抗治疗浓度在减低剂量的48 h内仍未达标，则每剂增加300 mg。仅在sC5b-9正常、维持血浆依库珠单抗治疗浓度和CH50<10％至少2周后，改为维持剂量和减量。维持治疗是将诱导剂量减低300 mg每周1次，维持4周。随后减量至最低剂量600 mg每周1次，维持2周。若补体sC5b-9正常，则从诱导剂量每周900 mg开始用药（图1）。由于依库珠单抗阻断补体通路，而补体在固有免疫对抗病原菌中发挥重要作用。依库珠单抗治疗aHUS过程中可能并发铜绿假单胞菌、脑膜炎球菌感染等，需引起注意[62]–[64]，可使用抗生素（环丙沙星、青霉素V等）预防依库珠单抗引起的感染[65]–[66]。依库珠单抗在回顾性小样本临床研究中取得良好疗效，然而仍需大规模、多中心、随机对照临床试验，以进一步验证其临床疗效。

**表3:** 依库珠单抗治疗造血干细胞移植相关血栓性微血管病（TA-TMA）的疗效

作者	年份	例数	其他治疗	随访时间	反应率（％）	总生存率（％）
Jodele等[67]	2014	6	TPE	−	66.7	33.3
Fontbrune等[68]	2015	12	TPE	14个月	50	33
Jodele等[61]	2016	18	−	1年	61	56
Bohl等[60]	2017	15	TPE，去纤苷+TPE，利妥昔单抗	30周	93	33
Rudoni等[69]	2018	10	TPE，免疫抑制剂	404（179~1259）d	70	60
Jan等[59]	2019	10	TPE，免疫抑制剂	90d	70	60
Schoettle等[70]	2019	6	血液透析	1年	67	78
Jodele等[9]	2020	64	无	1年	64	66

注：TPE：血浆置换

**图1:**
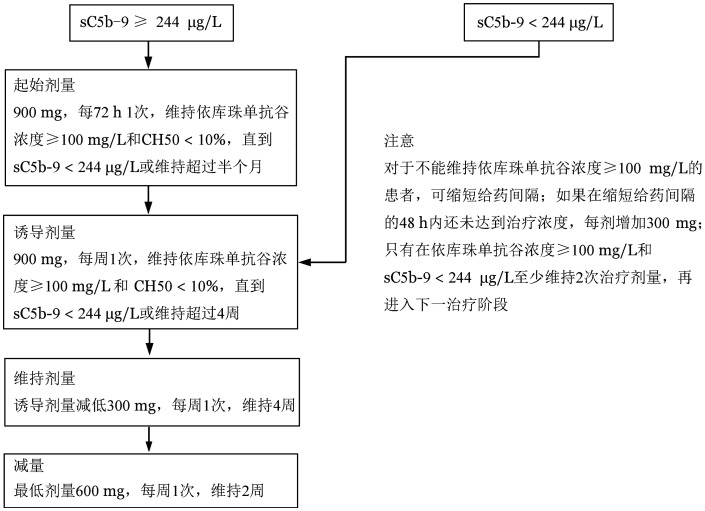
依库珠单抗治疗造血干细胞移植相关血栓性微血管病（TA-TMA）的用法[9] CH50：血清总补体

2. TPE：TPE可去除抑制ADAMTS13活性的相关抗体和补充缺少的ADAMTS13，是治疗TTP患者的一线疗法。然而，TA-TMA发病机制不同于TTP，TPE在TA-TMA治疗中的作用尚存在争议。TPE可能通过替换补体相关蛋白、移除抗补体因子H抗体和炎症因子等发挥对TA-TMA的治疗作用[7]。BMT-CTN共识统计1991至2003年期间的研究报道显示，TPE治疗TA-TMA的有效率为36.5％（0％～80％），死亡率高达80％（44％～100％），因此不建议将TPE纳入到治疗标准中[1]。然而，Laskin等[7]分析了2003至2010年TPE治疗TA-TMA的临床研究，显示中位有效率为59％（27％～80％）。2010年至今，TPE治疗TA-TMA的有中位有效率为59％（24％～100％）（表4）。TPE治疗合并GVHD的TA-TMA患者的有效率为22％[71]，疗效较单纯TA-TMA患者差。尽早应用TPE可能使TA-TMA患者获益[72]，然而，TPE不能改善TA-TMA肾脏预后[73]。由于缺少对照研究、TA-TMA严重程度不同及联合应用其他药物等，导致上述关于TPE疗效的研究异质性很大。TPE的干预时间、是否合并GVHD和TA-TMA发生的时间，可能影响TPE的疗效。支持治疗疗效欠佳的TA-TMA患者，应尽早进行TPE。建议采用新鲜冰冻血浆，每次置换量为1～1.5倍血浆容量（40～60 ml/kg体重），每天1次，持续1～2周。根据患者的临床表现、实验室指标和反应情况等缓慢减量。隔日1次，持续1～2周；继之每周2～3次，持续1～2周。如果减量过程中，实验室指标显示TA-TMA复发，则恢复到之前用量[6],[72]。如果没有条件实施TPE，可予新鲜冰冻血浆输注（10～20 ml/kg）改善症状。然而，短期内输注大量血浆可引起血容量增加，加重高血压症状或导致肺水肿甚至呼吸衰竭[74]。

**表4:** 2010年至今血浆置换（TPE）治疗造血干细胞移植相关血栓性微血管病（TA-TMA）疗效的文献汇总

作者	年份	例数	其他治疗	反应率	死亡率	其他发现
Kennedy等[71]	2010	11	无	27％	91％	TPE对合并GVHD的TA-TMA患者疗效较差
Jodele等[72]	2012	10	利妥昔单抗	90％	50％	尽早开始应用TPE可使TA-TMA患者获益
Mulay等[54]	2014	33	糖皮质激素、长春新碱、利妥昔单抗和达克珠单抗	24％	55％	迟发型较早发型TA-TMA患者应用TPE的疗效更好
Sartain等[73]	2019	15	依库珠单抗	100％	13％	TPE不能改善TA-TMA患者的肾脏预后

3. 利妥昔单抗：利妥昔单抗是一种抗CD20单克隆抗体，临床研究疗效证据较少。研究表明，利妥昔单抗用量为每周375 mg/m^2^，连续应用4周。Au等[75]报道，应用利妥昔单抗使5例移植后难治性TA-TMA患者的4例获得缓解。然而，所有患者的ADAMTS13水平较低，且1例患者伴有抗ADAMTS13抗体，提示该研究中的TMA患者中可能混杂有TTP，可能影响结果的评估。研究显示利妥昔单抗/TPE治疗TA-TMA患者的完全缓解率为67％[13]。

4. 去纤苷：去纤苷是一种从猪肠黏膜提取的单链寡聚脱氧核糖核苷酸复合物，能够稳定并保护内皮细胞，具有促纤溶、抗血栓形成、抗缺血、抗炎和抗粘附活性[76]–[77]。去纤苷用于治疗肝静脉闭塞症/肝窦隙阻塞综合征（VOD/SOS）、抗磷脂抗体综合征和TTP等[76],[78]。治疗TA-TMA的作用机制尚未完全阐明。多数研究中，去纤苷的使用剂量为20～40 mg·kg^−1^·d^−1^，维持治疗至少14 d。去纤苷治疗TA-TMA的有效率达65％～77％[79]–[81]。在临床应用中，去纤苷有良好的安全性，但仍需注意其出血风险[82]。

5. 其他：达克珠单抗（daclizumab）是一种人源抗CD25单克隆抗体，Wolff等[83]将钙调磷酸酶抑制剂替换为达克珠单抗，13例TA-TMA患者中11例获得缓解。长春新碱常用于淋巴瘤和白血病化疗及TTP的治疗，也被尝试用于TA-TMA的治疗。有学者报道，7例TA-TMA患者接受长春新碱单药或联合TPE治疗，6例获得完全缓解和长期生存[84]。此外，普伐他丁[85]、重组人可溶性血栓调节蛋白[86]在小样本临床研究中也显示出一定的疗效。

四、展望

TA-TMA的发病机制尚不明确，临床常被漏诊或延误诊断，死亡率很高。研究TA-TMA的发生机制、寻找新的早期生物学标志物或临床诊断指标为TA-TMA的早期诊断及靶向治疗提供理论基础，对改善TA-TMA患者预后具有重要意义。
